# Programming 2D/3D shape-shifting with hobbyist 3D printers†
†Electronic supplementary information (ESI) available. See DOI: 10.1039/c7mh00269f


**DOI:** 10.1039/c7mh00269f

**Published:** 2017-06-22

**Authors:** Teunis van Manen, Shahram Janbaz, Amir A. Zadpoor

**Affiliations:** a Additive Manufacturing Laboratory , Department of Biomechanical Engineering , Delft University of Technology (TU Delft) , Mekelweg 2 , Delft 2628CD , The Netherlands . Email: a.a.zadpoor@tudelft.nl ; Tel: +31-15-2781021

## Abstract

Materials and devices with advanced functionalities often need to combine complex 3D shapes with functionality-inducing surface features. Precisely controlled bio-nanopatterns, printed electronic components, and sensors/actuators are all examples of such surface features. However, the vast majority of the refined technologies that are currently available for creating functional surface features work only on flat surfaces. Here we present initially flat constructs that upon triggering by high temperatures change their shape to a pre-programmed 3D shape, thereby enabling the combination of surface-related functionalities with complex 3D shapes. A number of shape-shifting materials have been proposed during the last few years based on various types of advanced technologies. The proposed techniques often require multiple fabrication steps and special materials, while being limited in terms of the 3D shapes they could achieve. The approach presented here is a single-step printing process that requires only a hobbyist 3D printer and inexpensive off-the-shelf materials. It also lends itself to a host of design strategies based on self-folding origami, instability-driven pop-up, and ‘sequential’ shape-shifting to unprecedentedly expand the space of achievable 3D shapes. This combination of simplicity and versatility is a key to widespread applications.

## Abstract 2

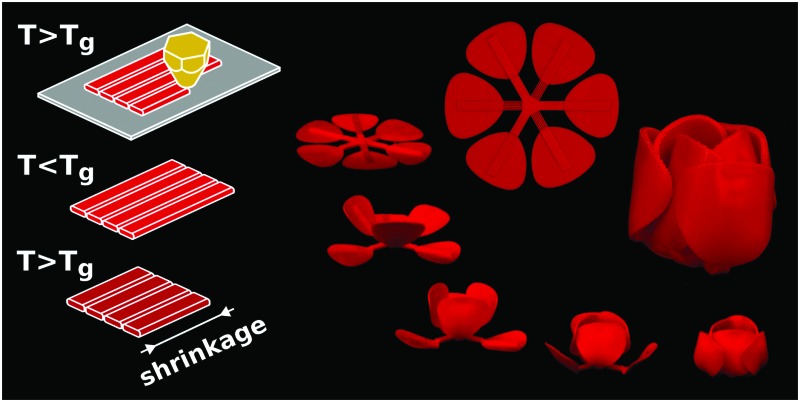
Fused deposition modeling (FDM) enables simultaneous programming and production of thermo-responsive shape-shifting materials.

##

Conceptual insights

We present a novel shape-shifting technique that requires only a hobbyist 3D printer and inexpensive off-the-shelf materials. Our approach is based on simultaneously printing and programming shape memory polymer materials in a fully automated and single-step production process. Upon triggering by high temperatures the printed planar construct changes its shape into a pre-programmed 3D shape. The presented technique lends itself to multiple design strategies that could be used alone and in combination with each other to achieve an unprecedented level of complexity in the final 3D shape. A particularly interesting design approach we demonstrate is the use of heat transfer concepts (through the incorporation of porosity and varying the thickness of the constructs) to program adjustable time delays, thereby enabling ‘sequential shape-shifting’. Shape-shifting materials are of significant interest as complex 3D shapes can be combined with precisely controlled surface functionalities such as bio-nanopatterns and printed electronics. Refined production techniques that are currently available for creating those surface features work only on flat surfaces. The presented shape-shifting technique offers a route to the production of those functionality-induced materials which have a broad range of applications in biotechnology, electronics and engineering of complex metamaterials.

## 

Materials that are programmed to develop 3D configurations from initially flat states propound a revolutionary approach with applications in biotechnology,^1^ electronics,^2^ and engineering of complex metamaterials.^3^ Combining passive and stimuli-triggered active layers into a multilayer construct is often used for programming the basic modes of shape-shifting such as bending and twisting.^4^–^8^ Swellable and shape memory polymers (SMPs) are widely used as active elements in such designs. Triggered by different stimuli such as an increase in temperature, humidity, or light, active elements can drive the shape-shifting process.^9^–^12^ Temporal shape transition of programmed SMP without any external stimulus is reported as well.^13^ Dimensional parameters and the programming procedure as well as the activation conditions can control the quality of the deformations.^8^ Complex 3D structures are then obtained through the rational arrangement of active elements in multi-ply constructs.^4^ For example, active hinges can control the folding state of origami.^5^,^14^ As an alternative to multi-layers, compressive stresses generated in swellable polymers could be used to induce complex shape-shiftings.^15^,^16^ The sequence of folding may be tuned to avoid locking and could be used as a tool to drive the folding kinematics of highly complex structures and improve the integrity of the target configurations.^17^,^18^

3D printing provides an alternative route to the programming of shape-shifting: the spatial arrangement of active (and passive) elements. In particular, sophisticated multi-material printing techniques could be used to combine different materials (*e.g.* hydrogel/polymer or polymer/polymer) to achieve multi-shape^19^ or reversible shape-shifting.^19^,^20^ Anisotropic addition of properties (*e.g.* the swelling ratio or stiffness) can include a fourth dimension into 3D printing techniques to achieve the so-called 4D printing.^21^,^22^ In a sharp contrast to the previously proposed techniques that require sophisticated or custom-made printers and spatial materials, here we present a versatile approach that requires no more than a hobbyist 3D printer and inexpensive off-the-shelf materials to implement many of the above-mentioned design routes and more.

Hobbyist 3D printers generally work on the basis of extrusion of polymeric filaments, a technology called fused deposition modeling (FDM) (Fig. 1a). During the printing process, the filament experiences temperatures above its melting temperature while being stretched. The high temperature allows for stretching and alignment of polymer chains along the direction of extrusion, which due to the constraints applied during the printing process (see the ESI,† document) is stored in the material as memory. Polylactic acid (PLA), a shape memory polymer with a melting temperature >180 °C, is the most widely used material in hobbyist FDM printers. 3D printed (*i.e.* extruded) PLA filaments simultaneously decrease in length and thicken once heated above their glass transition temperature, *T*_g_ (Fig. 1a). The percentage of the decrease in the filament length could be controlled through adjustment of the printing parameters including the extrusion and activation (*i.e.* triggering) temperatures as well as the layer thickness (Fig. 1c–e). The flat surfaces are made of a number of layers with small thicknesses (50–200 μm). We used layers with identical patterns as well as stacks of layers with different patterns (Fig. S2, ESI†) to fabricate flat surfaces that show different combinations of directional strains (*ε*_1_ and *ε*_2_) upon exposure to temperatures exceeding *T*_g_ (Fig. 1b). The negative planar strains are quite large (up to 0.27) while smaller positive strains (up to 0.06) are also possible (Fig. 1b).

**Fig. 1:**
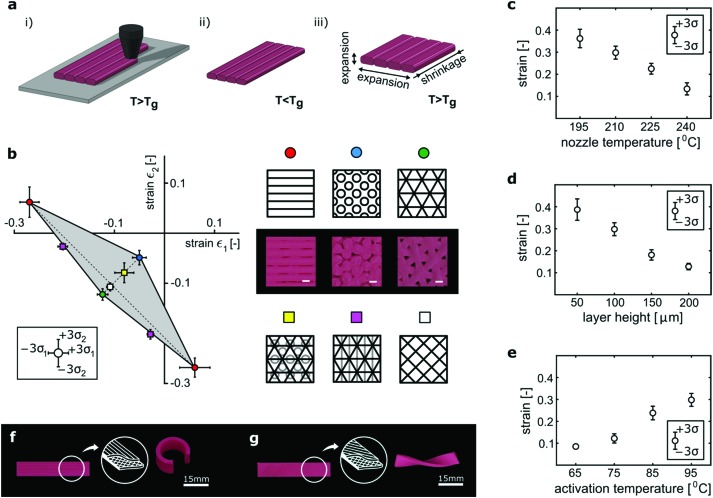
(a) As the filament is extruded during the printing process, it stretches and bonds with the previously printed layer. After cooling below *T*_g_, the stretching of the filament is stored as memory in the material. Upon heating above *T*_g_, the material relaxes, resulting in shrinkage in the longitudinal direction and expansion in both other directions. (b) The printing pattern of multi-ply square panels regulates their in-plane strains. The grey area shows the range of possible directional strains that we measured for multipattern panels. (c–e) Both printing and activation parameters control the shrinkage of multi-ply panels. (f) Basic shape-shifting in self-bending strips made by combining a longitudinal shrinking top layer with a semi-passive bottom layer. (g) Changing the orientation of the top layer results in self-twisting.

Flat surfaces (plies) with the desired deformation characteristics (*i.e. ε*_1_, *ε*_2_ duos) were then printed on top of each other for programming the desired shape transformation in the final multi-ply flat construct. The most basic mode of shape-shifting, *i.e.* a self-bending (self-rolling) strip, is achieved when a ply with relatively large anisotropic deformation characteristics (*e.g. ε*_1_ = –0.27, *ε*_2_ = 0.06) is printed on top of a ply with relatively small isotropic deformation characteristics (*e.g. ε*_1_ = –0.11, *ε*_2_ = –0.11) (Fig. 1f). The main straining direction of the ply with large deformation characteristics must be chosen to be in parallel to the length of the strip. The out-of-plane deformation occurs due to the resistance of the semi-passive ply against deformation of the active ply. If the main straining direction makes an angle with the length of the strip, a self-twisting (self-helixing) strip is made (Fig. 1g). Both self-bending and self-twisting elements were used as actuating elements in the shape-shifting designs described below.

Self-folding origami is one of the areas where semi-passive multi-ply panels with minimal deformation characteristics (*e.g. ε*_1_ = –0.11, *ε*_2_ = –0.11) that represent the rigid parts of the origami are combined with the actuating elements that exhibit large out-of-plane deformations (Fig. 2). The self-bending elements described above are the simplest types of such actuators. The width and thickness of the strips strongly affect the deformation characteristics (*e.g.* the radius of curvature, Fig. S4, ESI†) and could therefore be used to adjust the actuation behavior of the self-bending elements, thereby enabling a wide range of 3D shapes. Using multiple actuating elements – with different widths and thicknesses – in the same joint of a rigid origami not only allows for achieving different ultimate positions but also enables the separation of the actuation kinematics from the actuation kinetics (*i.e.* actuation force). A self-folding box is the simplest type of self-folding origami that can be made using the above-mentioned techniques (Fig. 2a). These design principles were found to be highly scalable and were, thus, applied to shapes with a larger number of folds and more complex topologies such as a self-folding dodecahedron (Fig. 2b). An example of a technically relevant 3D shape is the Miura-ori origami that has received much attention recently^23^,^24^ and is studied as a model of rigid origami. Manual folding of Miura-ori origami is challenging, making it difficult to perform extensive and consistent experiments. We combined two types of self-bending actuators, which bend in different directions, with semi-passive panels representing the rigid parts of the design to make a self-folding Miura-ori origami (Fig. 2c). Four actuators were used at each joint to increase the bending moment and overcome the locking caused by slight asynchronicities in the beginning of the bending of two or more actuators.

**Fig. 2:**
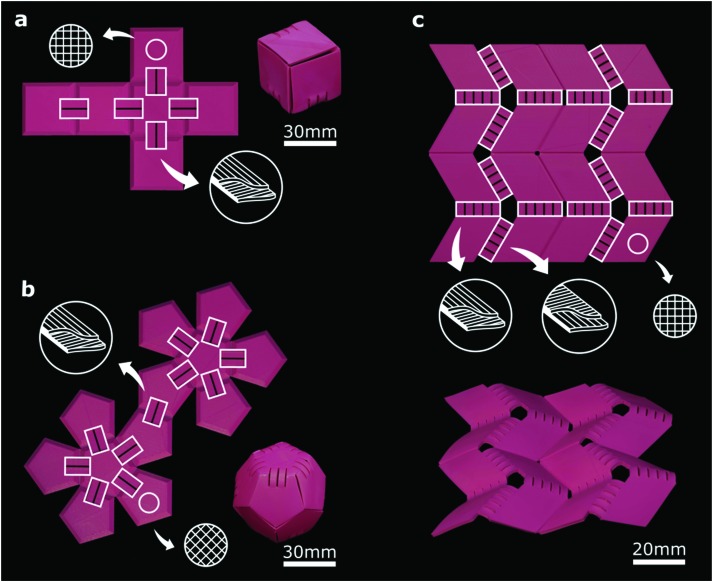
Self-bending elements (annotated in the figure as white rectangles) connect the semi-passive panels of origami structures. (a) A flat printed construct, consisting of 6 square panels connected by a thin layer to preserve the integrity of the structure, is folded into a cubic box upon activation (ESI,† Video 1). The printing patterns of the active bilayers and semi-passive panels are shown schematically. (b) Using the same method, a self-folding dodecahedron folds into its 3D shape (ESI,† Video 2). (c) The well-known Miura-ori folding pattern was printed and activated using two different types of self-bending elements (with different bending directions). The initially flat structure made of four Miura-ori units transforms its shape to the desired folded state upon activation (ESI,† Video 3).

Derivatives of the basic shape-shifting modes could be used for obtaining more complex shapes. For example, two narrow self-helixing strips connected by a number of semi-passive horizontal elements were used to fabricate a DNA-inspired structure (Fig. 3a).

**Fig. 3:**
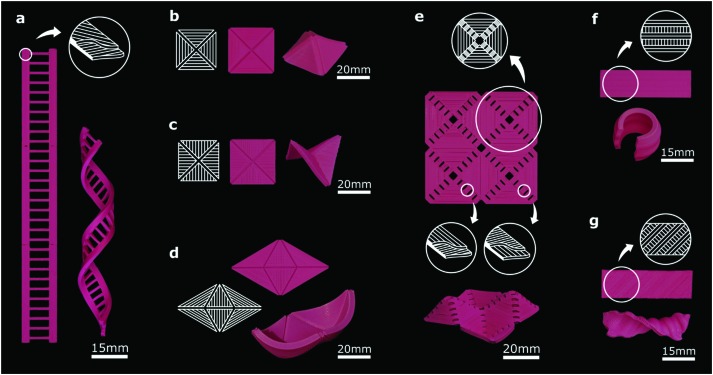
(a) Two initially flat self-twisting (bilayer) strands together with semi-passive connectors result in a DNA-inspired shape after activation (ESI,† Video 4). (b–d) The schematic of the monolayer panels with small grooves that were used to determine the folding lines. Compressive stresses generated by the anisotropic in-plane shrinkage of the panels result in out-of-plane buckling patterns that create the desired 3D shapes (ESI,† Videos 5 and 6). (e) Self-bending elements were placed at the folding lines of a shrinking panel to control the direction of buckling (*i.e.* upward *vs.* downward) (ESI,† Video 8). (f) Parallel arrangement of expanding and shrinking strips results in a monolayer self-rolling element (ESI,† Video 7). (g) Arrangement of the expanding and shrinking strips with a 45° angle with respect to the longitudinal direction results in a monolayer self-twisting element (ESI,† Video 7).

Every shape-shifting design demonstrated so far has been based on a combination of semi-passive panels with actuators. An alternative approach is to combine active panels that shrink in one direction and expand in another. In such a scenario, exposure to high temperatures creates compressive stresses that force some of the panels to buckle out of the plane. The active panels are connected *via* thin printed layers that act as joints and folding lines. The buckling caused by externally applied compressive forces has been recently proposed as an effective way for transforming 2D shapes to complex 3D shapes.^25^,^26^ The methodology we present here preserves the versatility of the buckling-induced 2D/3D shape transformations while taking it one step further by incorporating active elements, meaning that not only no external compressive forces are required but also that the number and complexity of the applied compressive forces could be drastically increased. To demonstrate some of the shapes that could be achieved through this approach, we designed self-folding pyramids (Fig. 3b), saddle shapes (Fig. 3c), and boats (Fig. 3d). To control the direction of the out-of-plane buckling, the thin connection between the panels could be replaced by some active bending elements. Using this approach, we assembled four pyramids (similar to Fig. 3b) of which, after activation, two popped-up in one direction and the other two in the other direction (Fig. 3e).

In an alternative approach to multi-ply panels, we used single-ply strips to induce out-of-plane shape-shifting. As opposed to the through-the-thickness arrangement of semi-passive and active plies used so far, the side-by-side alignment of shrinking and expanding monolayer strips can achieve self-bending (Fig. 3f) and self-twisting when the direction of the monolayer strips is oblique (Fig. 3g). After activation, parallel buckling lines appear on the surface of these monolayer strips as a consequence of energy minimization in their constituting micro-elements. This single-ply approach provides an additional shape-shifting avenue that could be potentially integrated into multi-ply designs to extend the space of achievable 3D shapes.

The most complex types of 2D/3D shape-shifting need to be performed in an ordered sequence of steps. In a self-folding origami, for example, some folds need to occur before others, necessitating the ability to program ‘sequential folding’. We used two strategies, namely variable porosity and variable thickness, to adjust the activation times of the active elements. Porosity was introduced to the panels by printing an array of grooves. We changed the depth and number of grooves to make panels with different porosities. Heat transfer occurs more readily in panels with higher porosity, which in turn results in faster activation. Thickness variation could be also used to adjust the time of activation, because it takes more time for the thicker panels to reach *T*_g_ and go into the soft glassy state. Activation will not commence before almost the entire thickness of the panel has reached the glass transition temperature, as the stiffness of the panel is much higher at low temperatures.^27^ Using both the above-mentioned strategies, we incorporated sequential folding into two designs. The first design imitates the gradual closing of the leaves of the shy (*i.e.* Mimosa pudica) plant (Fig. 4a). The second design (Fig. 4b) is a self-folding tulip with initially flat petals that sequentially fold to create multiple layers seen in natural tulips. When incorporated into the previously discussed design paradigms, time delays could tremendously enrich the programmability of initially flat constructs by enabling multiple shape-shifting steps.

**Fig. 4:**
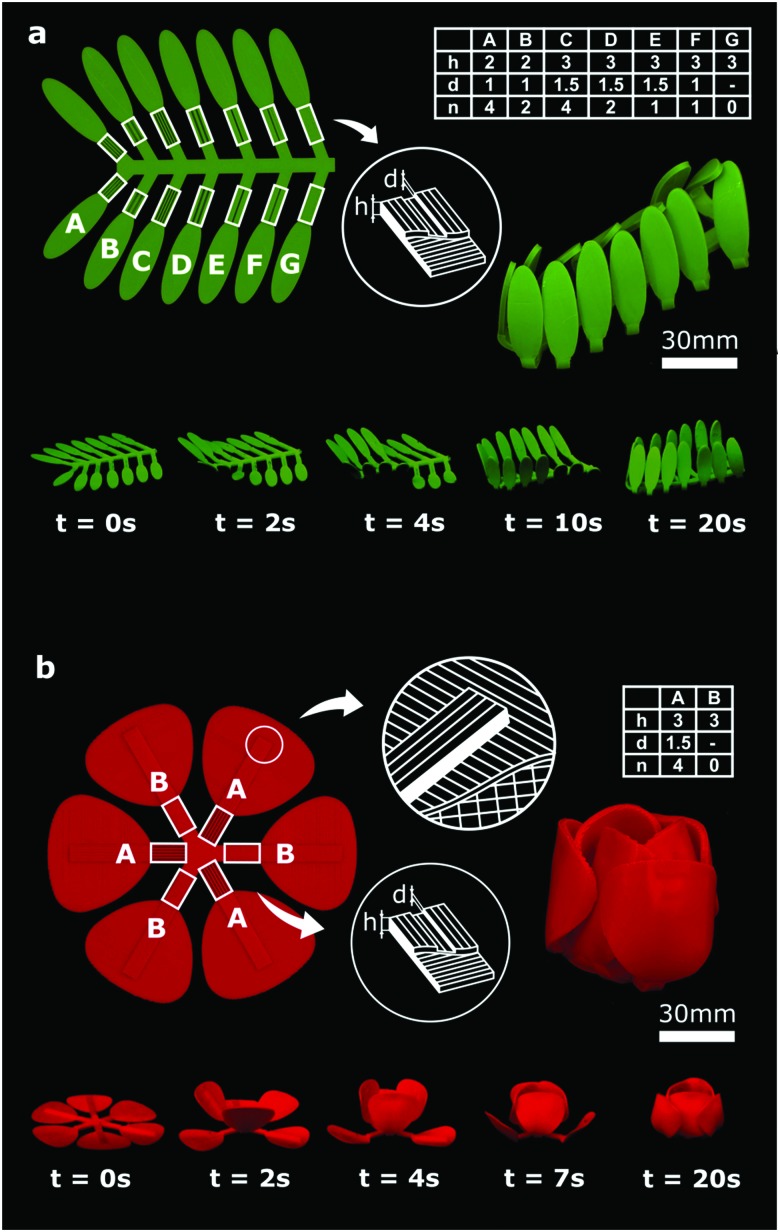
Sequential shape-shifting in two nature-inspired designs. The active regions in both structures are highlighted by white rectangles while the geometry and printing patterns are shown schematically. Dimensions (*h*: thickness, *d*: groove depth) and the number of grooves, *n* of the active elements are tabulated for both structures. (a) Gradual closure of the leaves of a structure resembling the shy plant. Sequential shape-shifting was controlled by the thickness and the dimensions of the grooves in the bilayer active elements (ESI,† Video 9). (b) Two-step folding of the initially flat petals to create a tulip (ESI,† Video 10). The time lapses illustrate the sequence of folding for both designs.

A number of points need to be discussed with respect to the shape-shifting behavior of the structures developed here. First, the results of the current study clearly show that different printing parameters result in different shape-shifting behaviors (Fig. 1b and Fig. S3, ESI†). Changing the printing parameters could, among other effects, result in an altered microstructure. Somewhat similar effects have been found in other studies.^28^ The micro-architectural parameters of the printed structures such as the porosity could also affect the shape-shifting behavior and were, for example, used for creating sequential folding. Second, the shape-shifting behavior of 3D printed PLA is activated by exposure to high temperatures. However, the homogeneity of the temperature field as well as the specific heat transfer conditions might be different based on the surrounding media. In addition, the other conditions of the surrounding environment such as humidity, pH, or liquid type could potentially affect the shape-shifting behavior by changing the microstructure of the printed material. Similar effects have been observed in other kinds of shape-shifting materials.^29^ Third, we used different in-plane arrangements of the 3D printed shrinking polymer to create shrinkage or expansion in lattice structures in response to the activation stimulus (*e.g.* Fig. S5b and c, ESI†). It would also be interesting to expand the current designs to create other kinds of negative/positive structural expansions similar to those reported in the literature.^30^ There are, however, some limitations such as the resolution of FDM printing and the layer-by-layer manner of printing that could limit the range of achievable structural expansion coefficients. Finally, although the approach presented here is aimed at achieving permanent shape-shifting, similar designs based on multiple responsive materials could be also used for programming reversible shape transformations.^31^

Given that a host of inexpensive 3D printers and PLA filaments similar to the ones used here are available in the market and that very complex 3D shapes could be achieved, there is no to little practical barrier to the widespread application of the proposed shape-shifting techniques. Advanced technologies that primarily work in 2D such as nanolithography, electron beam induced deposition,^32^ microarray printing,^33^ and direct-write electrospinning^34^ together with the functionalities they offer could then be easily incorporated into 3D devices. The design principles and fabrication techniques presented here could be transferred and scaled to serve as a general platform for programming the shape-shifting of flat materials and address the requirements of other printing technologies and materials.

## Author contributions

All authors participated (to different degrees) in the design of the experiments, interpreting the experimental results, writing the manuscript, and revising the manuscript for important intellectual content. TvM and SJ performed the experiments and analyzed the data.

## Supplementary Material

Supplementary information

Supplementary movie

Supplementary movie

Supplementary movie

Supplementary movie

Supplementary movie

Supplementary movie

Supplementary movie

Supplementary movie

Supplementary movie

Supplementary movie

Supplementary movie

Supplementary movie

Supplementary movie

Supplementary movie
