# Supplementary material for: Programming 2D/3D shape-shifting with hobbyist 3D printers
Source: Mater Horiz. 2017 Jun 22;4(6):1064–9. doi: 10.1039/c7mh00269f (PMC5735361; doi:10.1039/c7mh00269f)
Supplement: Supplementary file 1 [file MH-004-C7MH00269F-s001.pdf]

Electronic Supplementary Material (ESI) for Materials Horizons.
This journal is © The Royal Society of Chemistry 2017

Supplementary document to

Programming 2D/3D shape-shifting with hobbyist 3D printers

Teunis van Manen*, Shahram Janbaz*, Amir A. Zadpoor

Additive Manufacturing Laboratory, Department of Biomechanical Engineering, Delft University of Technology (TU Delft), Mekelweg 2, Delft 2628CD, The Netherlands.

1. General methods

3D printers (Ultimaker 2+, Ultimaker, The Netherlands) working on the basis of fused deposition modelling (FDM) and polyactic acid (PLA) polymeric filaments (Ultimaker, diameter = 2.85 mm, T_g = 60-65 ^oC) were used for fabrication of all designs presented in this work. PLA generally has good shape memory properties[1]. Samples were produced in the room temperature while the temperature of the glass print bed was set to 60 ^oC in order to improve the adhesion between the samples and the print bed. The PLA filaments were extruded through a standard 0.4 mm nozzle. A nozzle temperature of 210 ^oC, printing speed of 60 mm/s, filament flow of 2.1 mm^3/s, and layer thickness of 0.1 mm were used. Good adhesion between the fixed print bed and extruded material and the small gap between the nozzle and print bed, which result in swiping of the extruded filament by the moving nozzle tip, make sure that the melted material is stretched during printing. The stretched state of the melted filament will be frozen after cooling down and remains as memory in the body of the printed constructs. Should it not have been for the combined effects of swiping and material adhesion, no stress would have been accumulated and stored in the material. That was confirmed by a simple experiment in which PLA filaments extruded through the 3D printer nozzle using the same parameters as described above exhibited no memory.

The memory stored in the materials can be released by increasing the temperature above T_g while not constraining the printed structure at its boundaries. Printing patterns prescribe the directions of shrinkage and expansion into the body of printed constructs, which could be used as a tool for programming the shape-shifting of polymeric structures. A cubic glass water bath suitable for temperatures higher than T_g was used for activation of the printed structures, allowing us to track the deformations of the structures. The temperature of the water was controlled using a heating immersion circulator (CORIO CD, Julabo, Germany). Digital cameras were used to track the deformation of the studied specimens and a Matlab (Mathworks, version R2016b) code was used for image processing.

2. Effects of printing parameters on the directional strains of flat specimens

A number of test specimens (30×10×2 mm) were 3D printed with PLA filaments (color: silver). The extruded filaments were aligned in the longitudinal direction (the print path is shown in Figure S1a). The specimens were activated at 95 ^oC for at least 2 min to ensure the completion of the shape-shifting process. To provide free boundary conditions at both ends of the specimens, a weak connection was printed between the testing part of the specimen and its fixed end, which was attached to a gripper to keep the specimen in place (Figure S1b). The specimens were marked with black dots in order to easily track the change in length with image processing. The effects of nozzle temperature, printing speed, filament flow, and layer thickness were experimentally studied where each parameter was independently varied from the standard settings set by the manufacture (see Section 1). In addition, the effects of activation temperature were experimentally investigated.

---

* These authors contributed equally in this work.

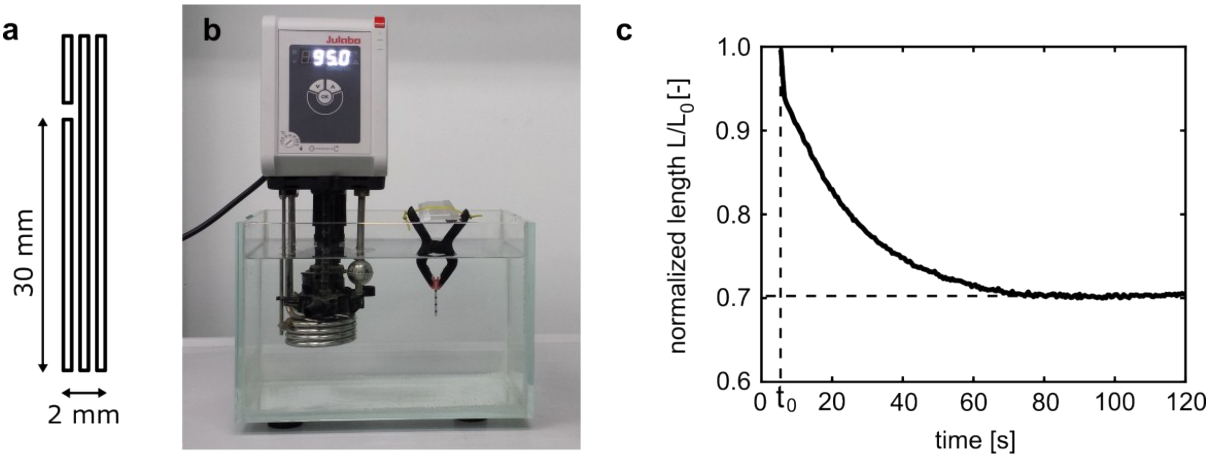

Figure S1. (a) The print path of each printed layer of the specimens. (b) The experimental setup used for activation of the specimens. The specimens were held by a gripper. (c) Change in the length of one sample specimen. The deformation starts at time t_0 after activation.

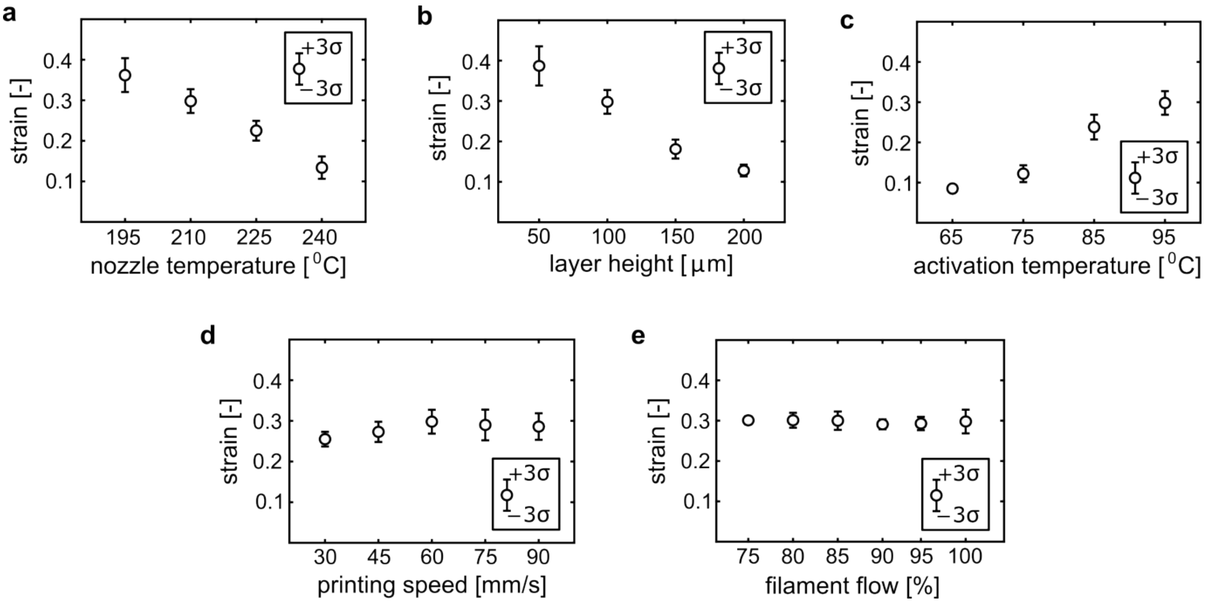

Figure S2. The effects of the printer settings and activation temperature on the shrinkage of the specimens. The average shrinkage over four tested specimens is plotted with the error bars representing three times the standard deviation from the mean.

The strain vs. time curves followed similar paths (Figure S1c) but with different onset times, t_0, and maximum strains. Three of the five studied parameters were found to highly influence the strain induced in the specimens (Figure S2). Both reduction in the nozzle temperature and layer thickness increase the maximum strain. The maximum strain could be increased from 13% to 36% by lowering the nozzle temperature from 240 ^{o}C to 195 ^{o}C (Figure S2a). Decreasing the layer thickness from 0.2 mm to 0.05 mm results in a 3-fold increase in the maximum strain (38% for 0.05 mm, Figure S2b). The activation temperature also strongly affected the contraction in the longitudinal direction (Figure S2c). The amount of shrinkage increased from about 13% to 29% when the activation temperature increased from 65 ^{o}C to 95 ^{o}C. The printing speed and filament flow did not significantly influence the shrinkage of the specimens (Figure S2d-e). The filament flow is expressed as a percentage of the standard material flow of 2.1 mm^3/s (at a printing speed of 60 mm/s).

The nozzle temperature highly influenced the shrinkage of the specimens. As the extruded filament cools down, some relaxation of the stretched polymeric chains happens before the

temperature drops below T_g. Printing at higher temperatures gives more time for relaxation and less stress will be stored inside the printed samples. Increasing the layer thickness has a similar effect, because it increases the cooling time. Moreover, reducing the layer thickness enhances the level of stretching of the polymer chains during the printing process.

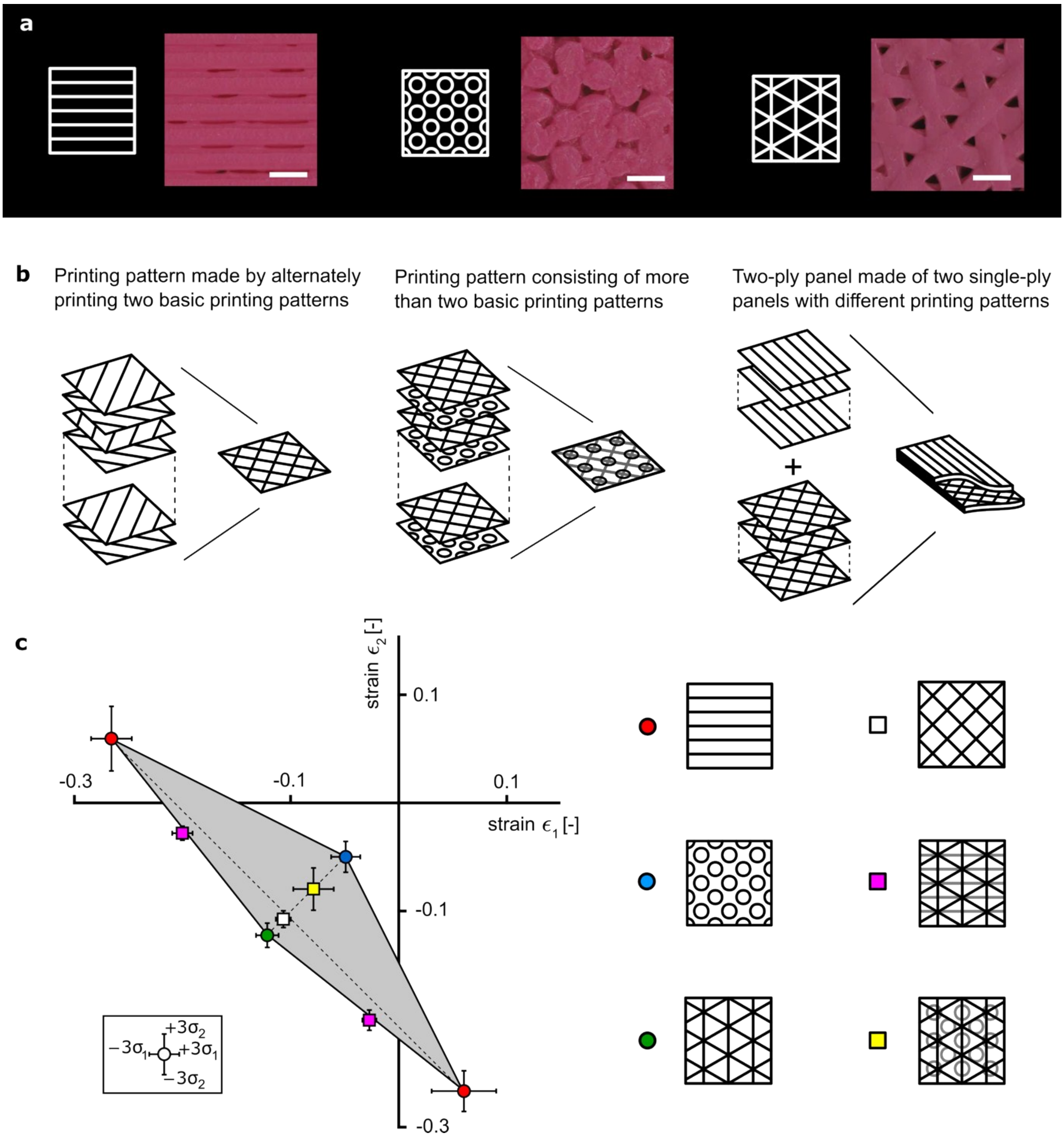

Figure S3. (a) Schematic representation and detailed images of the three basic printing patterns (scale bar = 0.5 mm). (b) Complex printing patterns and two-ply structures were made by combining basic patterns. (c) The effects of printing pattern on in-plane deformation. The average of four tested specimens is plotted and the error bars represent three times the standard deviation.

3. Effects of the printing pattern on the in-plane deformation of rectangular specimens

Different printing patterns were used for production of rectangular specimens (30×30×2 mm) from PLA filaments (color: silver). Three basic pattern designs, made by printing a stack of layers with identical patterns, were used (Figure S3a) together with a number of composite

patterns, which were produced by printing a stack made of layers with different patterns (Figure S3b). The standard printer settings (see Section 1 of this document) were used. Activation was done at 95 °C for at least 2 min to ensure shape-shifting was complete. The length and width of the specimens before and after activation were measured using a caliper.

The deformation duos (normalized change in the length of the specimens in both in-plane directions) are plotted in Figure S3c. The alignment of the filament in one direction resulted in 27% shrinkage and 6% expansion in both orthogonal in-plane directions. Both circular and triangular printing patterns showed isotropic in-plane shrinkage (5% and 12%, respectively). Alternating the printing direction between consecutive layers resulted in 11% shrinkage in both directions. Combining linear and triangular printing patterns resulted in highly anisotropic strains (ε_1 = -0.20, ε_2 = -0.03). On the other hand, the composite of triangular and circular printing patterns resulted in isotropic contractions equal to 8%.

A wide range of both isotropic and anisotropic in-plane deformation duos could be achieved by varying the printing pattern. Anisotropic deformations predominantly require layers with filaments aligned in one direction. Printing small circles severely limits the amount of stored deformation, reducing the shrinkage to only 5% in both directions. The triangular printing pattern shows the largest isotropic shrinkage of 12%. These three cases show some of the extremes of anisotropic and isotropic in-plane deformation that could be achieved by combining basic printing patterns into a composite pattern. The tested composite patterns showed in-plane deformations that were in-between those of the basic patterns they were made of.

The triangular pattern could be seen as a composite of three linear patterns rotated by 120°, although the triangular pattern is printed within one layer instead of printing three separate linear patterns on top of each other. However, the shrinkage is significantly different from the grid infill pattern (composite of two orthogonal linear patterns). The insets of Figure S3a show that the triangular pattern is more porous as compared with the grid infill pattern, which was confirmed by comparing the weights of both types of specimens. The average mass of a 30×10×2 mm specimen with triangular pattern and grid infill pattern were respectively 2.0 and 2.3 g. The larger porosity of the triangular infill pattern allows for more deformation (shrinkage) as compared with the grid pattern. Further increasing the porosity might result in larger deformations but will also reduce the stiffness of the panels.

4. Parametric study on two-ply strips

Two-ply specimens with a length of 30 mm were 3D printed from PLA (color: silver) to study the effects of dimensions (thickness, width) on their final shape. The printing patterns of the top and bottom plies are schematically shown in Figure S4a. The thickness of both plies was equal. Activation was done at 95 °C for at least 2 min to make sure the activation process was complete. One end of each specimen was fixed using a gripper and the free boundary was provided by a weak connection between the self-bending specimen and the fixed end. Marking the specimen with black dots allowed us to use image processing techniques for tracking the changes in the radius of curvature during the experiment.

Experimentally determined changes in the radius of curvature of one of the specimens is plotted in Figure S4b. The other specimens exhibited similar trends of deformation with the main difference being in their onset time, t_0, and minimum radius of curvature, $r_{\min}$. Both thickness and width of the specimens significantly influenced the shape-shifting of the two-ply specimens (Figure S4c). The results show radii of curvature in the range of 4.3 mm – 14.9 mm. The slender specimens (width = 5-10 mm) showed a 2-fold increase in their radius of curvature by increasing the thickness from 1.5 mm to 3 mm. Increasing the width from 5 mm to 10 mm did not change the results. Further increasing the width to 15 mm resulted in a 30% - 100% increase in the radius of curvature, dependent on the thickness.

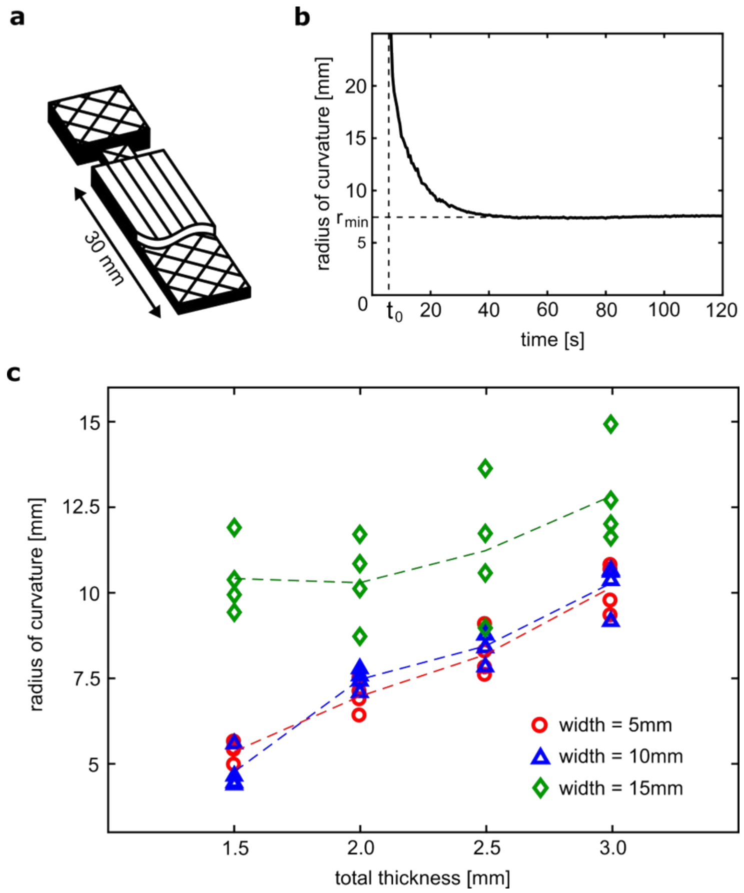

Figure S4. (a) Schematic representation of the two-ply specimen. A cut-out of the top layer is made to show the printing pattern of the bottom ply. (b) A sample curve representative of the tested specimens showing the change in the radius of curvature with time. The onset time of bending is denoted by t_0 and the minimum radius of curvature is denoted by r_{min}. (c) Experimental results show the radius of curvature of two-ply specimens with different sizes. Four specimens from each configuration were tested.

Increasing both the thickness and width of the two-ply strips resulted in larger radii of curvature after activation. According to the Timoshenko's bilayer bending model, the radius of curvature increases linearly as the thickness increases[2]. Fitting this linear relation between the radius and thickness to the experimental data showed that the experiments are in good agreement with the model for slender specimens ($R^2 = 0.99$ and $R^2 = 0.97$ for widths of 5 mm and 10 mm, respectively). By further increasing the width to 15 mm, the edge effects, which are not captured by the Timoshenko's bilayer model, started to become more prominent. The Poisson's effect causes the difference in the longitudinal strains of the top and bottom plies to be accompanied with an opposite difference in the transverse strains of both plies, resulting in the transverse bending of the two-ply strips. This double curvature increases the bending moment of inertia in the longitudinal direction, thereby increasing the radius of curvature.

5. Shape-shifting of cellular structures

Cellular 2D structures were printed from PLA (color: magenta) using the standard printing settings. All specimens were produced with an edge thickness of 3.2 mm and a height of 7.5 mm. Two mechanisms of shape-shifting in the cellular structure were used to program both 2D to 2D and 3D to 3D shape-shifting. Printing of a 5×1 square array was performed with filament extrusion along the edge to program reduction in the edge length or in transverse direction to program increase in the edge length (Figure S5a). The programmed expansion

and shrinkage of the edges results in the bending of the structure. Alternative to programming the change in the length of the edges, bending of the edges can be programmed by printing an expanding and shrinking strip side-by-side in one edge. This method is illustrated by a 3 x 3 array which shows pattern transformation from rectangular cells to hour-glass-shaped cells (Figure S5b). Expansion of a 2×2 cellular structure is achieved by straightening some initially curved edges using the same method (Figure S5c). 3D to 3D shape-shifting is illustrated with a cellular cubic structure made through manual assembly of 2D cellular unit-cells, which shows volumetric expansion upon activation (Figure S5d). Both the 5×1 and 3×3 square arrays were activated at 95 °C while other cellular structures were activated at 85 °C to avoid excessive deformations. Movies capturing the shape-shifting of the cellular structures were recorded and can be found as electronic supplementary material.

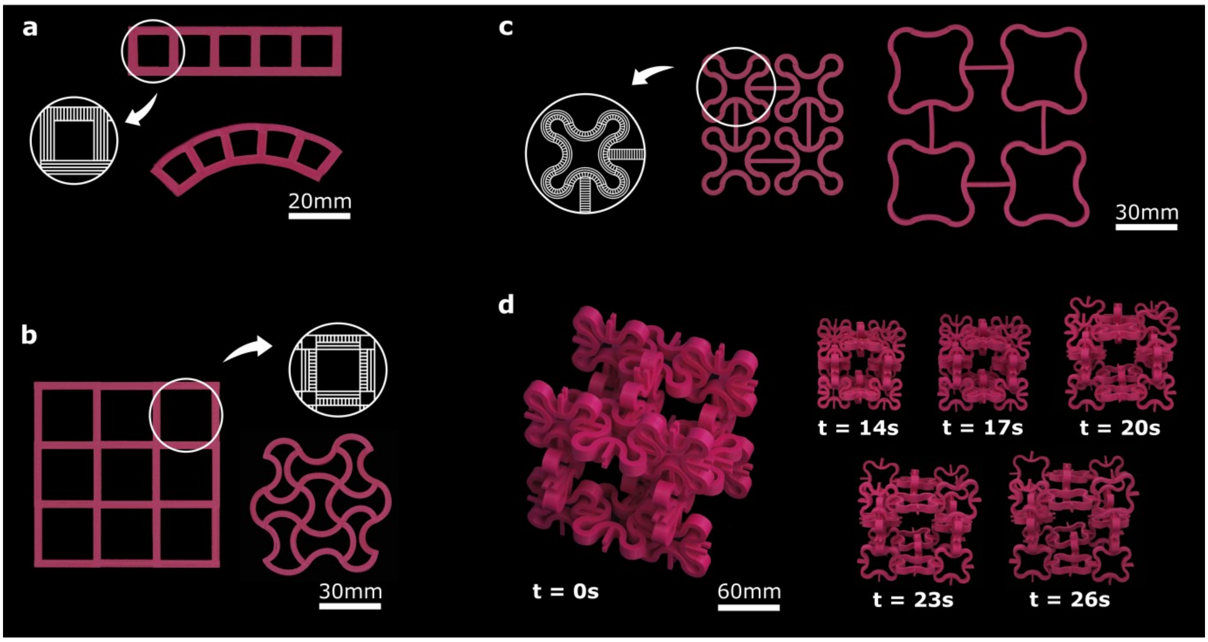

Figure S5. 2D to 2D and 3D to 3D shape-shifting of cellular structures. (a) Bending of a 5×1 square array upon activation (supplementary video 11). (b) Pattern transformation from a 3×3 square array to a 3×3 hourglass-shaped array (supplementary video 12). (c) A 2×2 expanding cellular structure (supplementary video 13). (d) The volumetric expansion of an assembly of 20 expanding unit-cells (supplementary video 14). Unit-cells with the same printing patterns as the expanding structure (Figure S5c) were used.

6. Supplementary movies

Video 1: Rigid panel origami: Folding of a box.
Video 2: Rigid panel origami: Folding of a regular dodecahedron.
Video 3: Rigid panel origami: Folding of an array of Miura-ori patterns.
Video 4: A DNA-inspired structure.
Video 5: Out-of-plane buckling of 3D structures driven by shrinkage and expansion of the panels.
Video 6: Out-of-plane buckling of a boat-like construct driven by the shrinkage/expansion and curving of the panels.
Video 7: Bending/twisting of a monolayer strip driven by its in-plane shrinkage/expansion.
Video 8: 4 pyramids pop-up in different directions controlled by active hinges.
Video 9: Gradual closing of the leaves of the shy plant.
Video 10: Sequential folding of a tulip.

Video 11: Bending of a 5×1 array driven by expansion and shrinkage of the edges.
Video 12: Pattern transformation of a 3×3 square array from rectangular cells to hour-glass-shaped cells.
Video 13: Expansion of a 2×2 cellular array by straightening of the initially curved edges.
Video 14: Volumetric expansion of an assembly of 20 expanding unit-cells.

7. Printing and activation parameters

Table S1. The printing parameters and activation conditions which were used for production and activation of the designs presented in this work.

**Structure**	**Figure**	**Printing speed [mm/s]**	**Nozzle temperature [^{0}C]**	**Layer height [μm]**	**Activation temperature [^{0}C]**
Box	2a	60	210	100	95
Dodecahedron	2b	60	210	100	95
Miura-ori	2c	60	210	100	95
DNA	3a	60	210	100	95
Pyramid	3b	60	210	100	95
Saddle	3c	60	210	100	95
Boat	3d	60	210	100	95
4 pyramids	3e	60	210	100	95
Bending strip	3f	60	195	50	95
Twisting strip	3g	60	195	50	95
Mimosa pudica	4a	60	210	100	90
Tulip	4b	60	210	100	90
5 x 1 array	S5a	60	210	100	95
3 x 3 array	S5b	60	210	100	95
2 x 2 array	S5c	60	210	100	85
3D cellular structure	S5d	60	210	100	85

References

1 Senatov, F. *et al.* Mechanical properties and shape memory effect of 3D-printed PLA-based porous scaffolds. *Journal of the mechanical behavior of biomedical materials* **57**, 139-148 (2016).
2 Timoshenko, S. Analysis of bi-metal thermostats. *JOSA* **11**, 233-255 (1925).
